# A Scoring System Contributing to the Detection of Radiation Pneumonitis in Elderly Patients with Lung Cancer—Results of the POLCAR Trial

**DOI:** 10.3390/cancers18182973

**Published:** 2026-09-14

**Authors:** Dirk Rades, Inga Zwaan, Elisa Marie Groh, Cansu Delikanli, Daphne Schepers-von Ohlen, Laura Doehring, Sabine Bohnet, Charlotte Kristiansen, Hanne Falk Grauslund, Christian Felix Schulz, Stefan Janssen

**Affiliations:** 1 Department of Radiation Oncology, University Medical Center Schleswig-Holstein, Luebeck Campus, 23538 Lübeck, Germany; inga.zwaan@uksh.de (I.Z.); elisamarie.groh@uksh.de (E.M.G.); cansu.delikanli@uksh.de (C.D.); daphne.schepers-vonohlen@uksh.de (D.S.-v.O.); laura.doehring@uksh.de (L.D.); 2 Department of Radiation Oncology, University of Luebeck, 23562 Luebeck, Germany; stefan.janssen@uksh.de; 3 Department of Pulmonology, University Medical Center Schleswig-Holstein, Luebeck Campus, 23538 Lübeck, Germany; sabine.bohnet@uksh.de; 4 Department of Oncology, Vejle Hospital, University Hospital of Southern Denmark, 7100 Vejle, Denmark; charlotte.kristiansen@rsyd.dk (C.K.); hanne.falk.grauslund@rsyd.dk (H.F.G.); 5 Department of Radiation Oncology, University Medical Center Schleswig-Holstein, Kiel Campus, 24105 Kiel, Germany; christian.schulz@uksh.de; 6 Medical Practice for Radiotherapy Rundestrasse, 30161 Hannover, Germany

**Keywords:** lung cancer, radiotherapy, radiation pneumonitis, scoring system, Youden index

## Abstract

Background/Objectives: A symptom-based scoring system (0–9 points) for the identification of radiation pneumonitis was previously evaluated in lung cancer patients without age restrictions. A threshold of five points appeared optimal to detect pneumonitis. Since elderly patients have a higher pneumonitis risk, this threshold may not be valid for this group. The POLCAR trial evaluated performance and threshold selection in elderly patients. Methods: Diagnostic discrimination was assessed by receiver operating characteristic analysis. The areas under the curve (AUCs) and a null hypothesis that AUC equaled 0.7 were evaluated. Youden indices were used to identify candidate thresholds. In addition, the change from baseline and patient satisfaction were assessed. The dissatisfaction rate should be <20%. Results: Thirty new patients, plus 31 patients from another prospective trial with an almost identical design, qualified for analyses. The AUC for the pneumonitis score was 0.85 (95% confidence interval: 0.73–0.97). The highest Youden index (0.535) was observed for a threshold of four points. A threshold of five points led to a similar Youden index, a lower sensitivity, and higher specificity. An increase from the baseline by two points had a Youden index of 0.566 and a sensitivity of 100%, while an increase by three points had a Youden index of 0.550 and a specificity of 92.5%. Statistically superior discrimination for the change-from-baseline approach was not observed. The dissatisfaction rate was 5.4%. Conclusions: The scoring system showed good discrimination and high acceptance by patients. A threshold of four points appeared appropriate use in routine screening for the early detection of pneumonitis, whereas the change from baseline was considered useful as an exploratory tool when baseline data were available. Since only eight pneumonitis events occurred, threshold estimates require external validation.

## Simple Summary

The early detection of pneumonitis after radiotherapy for lung cancer is clinically important and may be supported by structured scoring systems. Such a system was evaluated but may not be suitable for elderly patients who generally have a higher risk of pneumonitis. The POLCAR trial evaluated a scoring system in patients aged ≥ 65 years who received radiotherapy for lung cancer. The score showed good discrimination ability (AUC > 0.8) and high patient satisfaction (94.6%). The highest Youden index was observed for a threshold of four points. Moreover, when considering the change from baseline, an increase in two points provided the highest corresponding Youden index but required a valid baseline score. A threshold of four points appeared to be most suitable for routine screening for the early detection of pneumonitis in elderly patients, as it was found to be easy to apply and did not require a baseline reference value.

## 1. Introduction

Radiotherapy is a common treatment for locally advanced lung cancer [1]. It can lead to radiation pneumonitis that mainly occurs after completion of the radiotherapy course, and may occur up to 5 months following treatment [2,3,4,5,6]. In the era of consolidation systemic treatment for locally advanced lung cancer—including check-point inhibitors, such as the programmed death-ligand 1 (PD-L1) inhibitor durvalumab, and tyrosine kinase inhibitors, such as the epidermal growth factor receptor (EGFR) inhibitor osimertinib—the risk of pneumonitis has further increased [7,8,9,10]. Free radicals result in the damage of DNA of both type I and type II pneumocytes. This process induces apoptosis, the release of pro-inflammatory mediators, and an inflammatory reaction conveyed by an infiltration of different cell types [2,3]. Subsequently, disruption of the alveolarcapillary barrier and the loss of surfactant occur, which leads to the malfunction of type II pneumocytes, subsequent interstitial edema, and finally, a collapse of the alveoli [3]. Radiation pneumonitis can become a serious complication and may even lead to treatment-related death [11].

Since the latency period between the end of radiotherapy and the onset of pneumonitis may be quite long, the leading symptoms of this subacute complication, i.e., shortness of breath, cough, and temperature, may be misinterpreted [12]. In particular, patients may be erroneously treated with antibiotics instead of corticosteroids. Twenty-five years ago, Inoue et al. reported that patients with lung cancer who developed severe radiation pneumonitis had a significantly worse 3-year survival rate than patients with no or mild radiation pneumonitis [13]. In their study, 94 out of 191 patients developed pneumonitis following thoracic irradiation, which was mild in 69 patients and severe in 25 patients. The 3-year survival rates were 33.4% without pneumonitis, 38.2% in patients with mild pneumonitis, and 0% in patients with severe pneumonitis. Thus, it is crucial to identify pneumonitis as early as possible. Since the patients are only seen every few weeks by a pulmonologist or radiation oncologist after they have completed their course of radiotherapy, pneumonitis may be detected significantly later than desired. Its identification may be accelerated by an app that can be utilized by the patients themselves.

An important contribution to such an app is the development of a scoring system that allows for discrimination between a high probability of radiation pneumonitis and no pneumonitis. In particular, the most appropriate cut-off score with respect to sensitivity and specificity is required. In a previous prospective study, we were able to provide the optimal cut-off score to detect symptomatic (grade ≥ 2) radiation pneumonitis in a cohort of patients without an age limit [6,14]. Of the scores ranging from zero to nine points, a score of five points was found most appropriate to identify grade ≥ 2 radiation pneumonitis. In addition, an increase by at least three points from the pre-radiotherapy score (baseline) was shown to be an indicator of pneumonitis.

The number of elderly patients with lung cancer is constantly growing. And these patients are known to have an increased risk of developing radiation pneumonitis [15,16,17]. Therefore, it was discussed whether the previously identified optimal cut-off score might also be valid for elderly lung cancer patients. These considerations have led to the present POLCAR trial [18]. Its primary objective was the definition of the most appropriate cut-off score for the identification of radiation pneumonitis. Moreover, the roles of the increase in scores from baseline and of patient satisfaction with the scoring system were evaluated. The findings of this study will contribute to the earlier detection of radiation pneumonitis and to the creation of an app that elderly lung cancer patients can use themselves at home, both during and after completion of their radiation treatment.

## 2. Materials and Methods

The initial protocol of the POLCAR trial was approved by the responsible ethics committee at the University of Lübeck on the 21 June 2024 (file 2024-315_01). Moreover, the POLCAR trial was submitted to clinicaltrials.gov and first posted on the 28 June 2024 (identifier: NCT06480734). An amendment to the protocol was approved by the ethics committee in Luebeck on the 14 March 2025 (file 2024-315_02). According to the amendment, it was possible to include patients from a previous prospective trial (PARALUC) with an almost identical design who were treated in institutions participating in the current POLCAR trial and met the eligibility criteria of the POLCAR trial [6]. These criteria included pathologically confirmed diagnosis of non-small-cell (NSCLC) or small-cell (SCLC) lung cancer, indication for radiotherapy with or without systemic treatment, a mean dose to the affected lung of more than 13 Gy, aged 65 years or older, written informed consent given by the patient, and patient’s ability to consent. Patients with expected non-compliance or a baseline score of more than 4 points (Table 1) were not considered eligible. Patients with a baseline score of 5 or more points (Table 1) were supposed to be unable to appropriately tolerate the required total radiation dose.

During the course of radiotherapy, which took 6 to 7 weeks in all but two patients, the study participants had an appointment with a treating radiation oncologist every week. During the visit, they indicated the presence and severity of cough, the shortness of breath (dyspnea), and a temperature (Table 1). After completion of the radiotherapy course, the symptoms were queried via telephone, scheduled for a period of 24 weeks. If a patient’s score showed an increase of 2 points in comparison to the baseline score, the patient was asked to come to the treating institution to receive additional diagnostic procedures (lung function test, computed tomography), if considered necessary [15,16]. In case of grade ≥ 2 pneumonitis, corticosteroids (prednisolone) were administered for at least 3 months [4,14].

To qualify for the analyses of the POLCAR trial, a patient must have completed at least 10 symptom questionnaires (including baseline). One patient only gave informed consent during week 6 of the radiotherapy course. Since regular baseline assessment and questionnaires of weeks 1 to 5 were not available, this patient was rated as screening failure. Of the remaining 32 new patients recruited for the POLCAR trial, 30 patients could be included in the analyses with respect to primary and secondary objectives. Two patients died before completion of the required 10 symptom questionnaires. Of the 57 patients of the previous PARALUC trial, 31 patients qualified for the analyses of the POLCAR trial. Twenty-two patients were younger than 65 years, three patients completed less than 10 symptom questionnaires, and one patient received a hypo-fractionated radiation boost [6].

Thus, the data of a total of 61 patients, 23 females and 38 males, were included in the analyses of the present POLCAR trial. The median age was 73 years (range: 65–88 years). The median total radiation dose was 62 Gy (range: 50–68 Gy). Fifty-seven patients were treated with volumetric-modulated arc therapy, and four patients in intensity-modulated radiation therapy. Prior to the start of irradiation, 28 patients had received systemic treatment, mainly platin/etoposide with or without immunotherapy (nine patients), platin/vinorelbine with or without immunotherapy (seven patients), or platin/pemetrexed with or without immunotherapy (seven patients). During the course of radiotherapy, systemic treatment was administered to 52 patients, most frequently with carboplatin/paclitaxel (18 patients), platin/vinorelbine (11 patients), platin/etoposide (eight patients), or platin/pemetrexed (seven patients). Five patients did not receive systemic treatment prior to or during radiotherapy. The distribution of patient-, cancer-, and treatment-related characteristics is shown in Table 2.

### Statistical Methods

The primary analysis population comprised all patients with documented radiation pneumonitis status and at least 10 completed symptom questionnaires. For patients who developed radiation pneumonitis, the score recorded at pneumonitis diagnosis was used; for patients without pneumonitis, the maximum score observed during and following the radiotherapy course was used. This patient-specific maximum score or score at the time of pneumonitis constituted the primary endpoint. Categorical variables were summarized as frequencies and percentages. Continuous variables are reported as medians with interquartile ranges and as means with standard deviations. Comparisons between patients with and without radiation pneumonitis were performed using two-sided Wilcoxon rank-sum (Mann–Whitney) tests with tie correction. Risk-factor analyses were exploratory; no adjustment for multiplicity was performed.

Diagnostic performance of the maximum score/score at pneumonitis was assessed using receiver operating characteristic (ROC) analysis. Areas under the ROC curve (AUCs), corresponding standard errors, and 95% confidence intervals were estimated using non-parametric methods based on an established approach [20]. The null hypothesis that the AUC equaled 0.70 was evaluated using a Wald-type normal approximation based on the DeLong standard error. Analyses were performed using the SAS LOGISTIC procedure with ROC and ROCCONTRAST statements. The benchmark of 0.70 was based on a classification of accuracy of a diagnostic test, where an AUC of >0.7 is rated as better than poor, an AUC > 0.8 as good, and an AUC > 0.9 as excellent. A two-sided significance level of 5% was applied. As an exploratory analysis, the same ROC-based methodology was applied to change from baseline to maximum or pneumonitis-associated score (max_diff). These analyses were supportive and hypothesis-generating.

For both endpoints, sensitivity, specificity, positive predictive value, negative predictive value, accuracy, and the Youden index were calculated across all observed integer thresholds. The Youden-optimal threshold was defined as the cut-off maximizing sensitivity plus specificity minus 1 [21,22]. Because thresholds were selected from the same dataset in which performance was estimated, all cut-off-specific operating characteristics should be interpreted as exploratory and require external validation.

To evaluate the relationship between symptom burden and pneumonitis risk, patients were categorized into tertiles of the maximum score/score at pneumonitis. Pneumonitis incidence across ordered tertiles was assessed using an exact Cochran–Armitage trend test, which is the appropriate ordered-trend test for a binary outcome across ordered score groups. Prespecified exploratory risk-factor analyses evaluated baseline demographic and clinical characteristics, including gender (female vs. male), age group (≤73 vs. >73 years, median age: 73 years), Eastern Cooperative Oncology Group performance status (ECOG 0–1 vs. 2–3), body-mass index (BMI < 30 vs. ≥30 kg/m^2^), primary tumor stage (T 1–2 vs. 3–4), lymph node status (N 0–1 vs. 2–3), distant metastases (M0 vs. 1), American Joint Committee on Cancer (AJCC) stage (I–II vs. III–IV), histology (NSCLC vs. SCLC), upfront resection (no vs. yes), systemic treatment prior to and/or during the course of radiotherapy (no vs. yes), consolidation systemic treatment (no vs. yes), total radiation dose (≤60 vs. >60 Gy), pre-radiotherapy Forced Expiratory Volume in 1 s (FEV1 < 1.6 vs. ≥1.6 L), significant cardiovascular disease (no vs. yes), chronic obstructive lung disease (no vs. yes), and autoimmune disease (no vs. yes). Thirty patients received systemic consolidation treatment following radiotherapy with check-point inhibitors or tyrosine kinase inhibitors, which included durvalumab in 24 patients, cemiplimab in one patient, azetolizumab in one patient, nivolumab in one patient, pembrolizumab in one patient, osimertinib in one patient, and durvalumab followed by pembrolizumab in one patient. Associations with radiation pneumonitis were assessed using Fisher’s exact tests. For each subgroup, the number and proportion of patients who developed radiation pneumonitis were reported. Graphical displays included ROC curves, score distribution histograms, and comparative box plots stratified by pneumonitis status. Histograms were displayed as percentages. Box plots were accompanied by the same two-sided Wilcoxon rank-sum *p*-value reported in the descriptive score table. All analyses were performed using SAS software, version 9.4 (SAS Institute, Cary, NC, USA).

In addition, patient satisfaction with the scoring system was evaluated after completion of the radiotherapy course. Patients completed a questionnaire modified according to Schrepp et al. [23] that consisted of four items. For each item, the patients could award 1 to 7 points to indicate whether the scoring system was comprehensible, provided a feeling of safety, was easy to use, and was helpful. Higher scores indicated a higher level of satisfaction. The maximum achievable score was 4 × 7 = 28 points. In accordance with the previous PARALUC trial, a mean score < 4.0 points indicated dissatisfaction with the scoring system [6]. If the dissatisfaction rate in the entire cohort was greater than 20%, the scoring system was regarded as not useful in its present form. If the rate exceeded 40%, the tool was regarded not useful at all. Moreover, mean scores and standard deviations of the four items representing patient satisfaction with the scoring system were calculated.

## 3. Results

**Figure 1:**
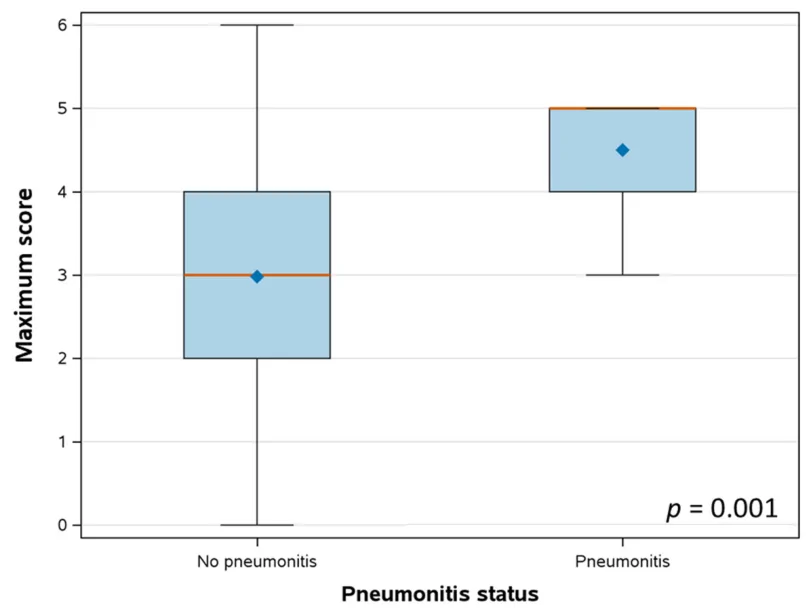
Comparative box plot showing the distribution of maximum scores in patients who developed pneumonitis and those without pneumonitis. The blue rhombus represents the mean score.

**Figure 2:**
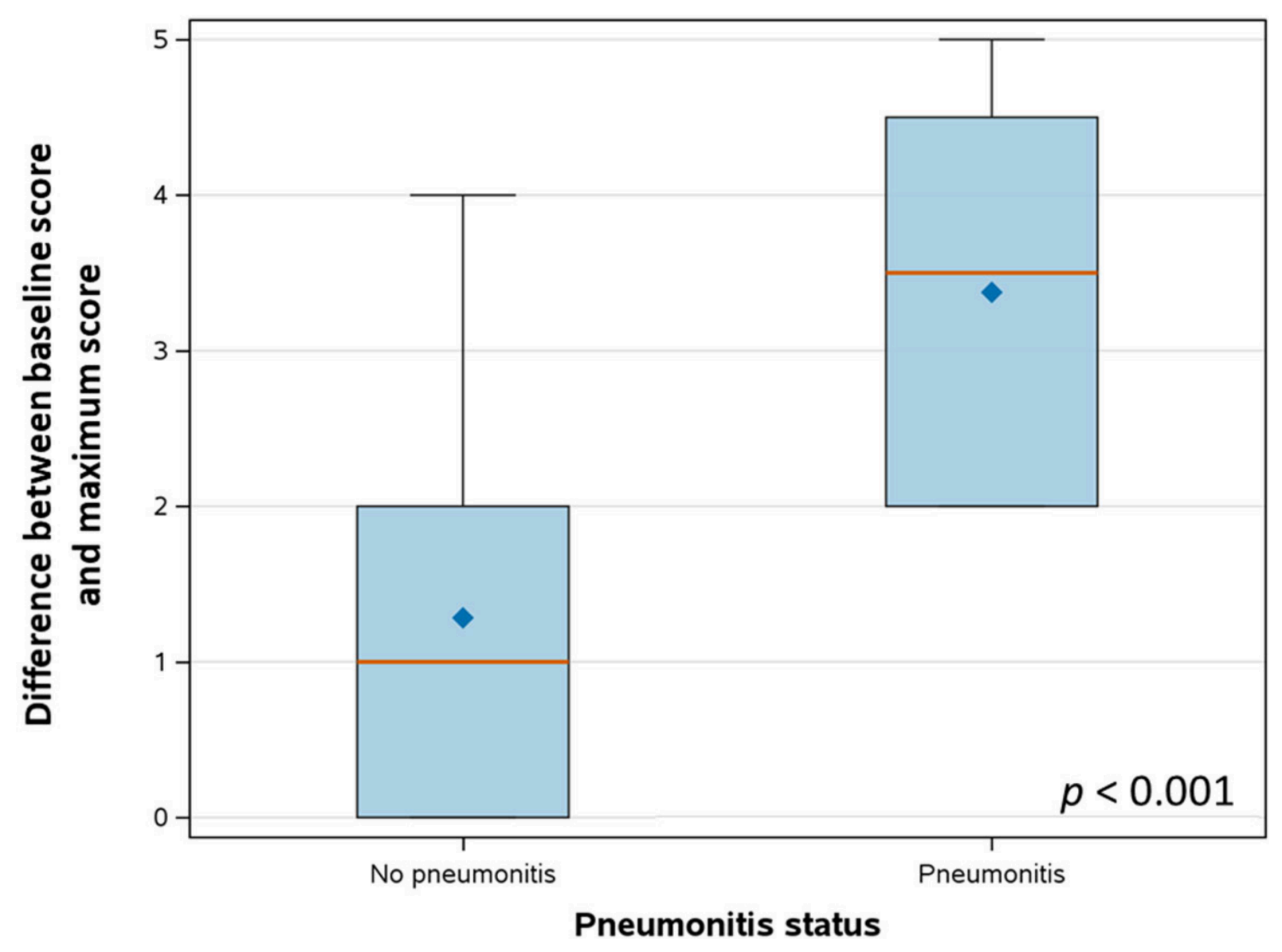
Comparative box plot showing the distribution of the difference between baseline scores and maximum scores in patients who developed pneumonitis and patients without pneumonitis. The blue rhombus represents the mean difference.

Among the 61 patients included in the primary analysis set, eight patients (13.1%) developed radiation pneumonitis after median 12.5 weeks (range: 1–19 weeks) following radiotherapy. The baseline scores did not differ significantly between patients who developed pneumonitis and those without pneumonitis. In contrast, the mean and median maximum scores were higher in patients developing pneumonitis than in patients without pneumonitis (Figure 1), and the increase from baseline was more pronounced (Figure 2). The corresponding figures are summarized in Table 3.

When stratifying pneumonitis by tertiles of the maximum scores or scores at the time of pneumonitis, the corresponding rates in the tertiles of 0–2 points (19 patients), three points (17 patients), and 4–6 points (25 patients) were 0.0% (no patient), 5.9% (one patient), and 28.0% (seven patients), respectively (*p* = 0.005). The maximum score showed good discrimination ability with an AUC of 0.85 (95% confidence interval: 0.73 to 0.97), exceeding the prespecified benchmark of 0.70 (*p* = 0.018) [24].

**Figure 3:**
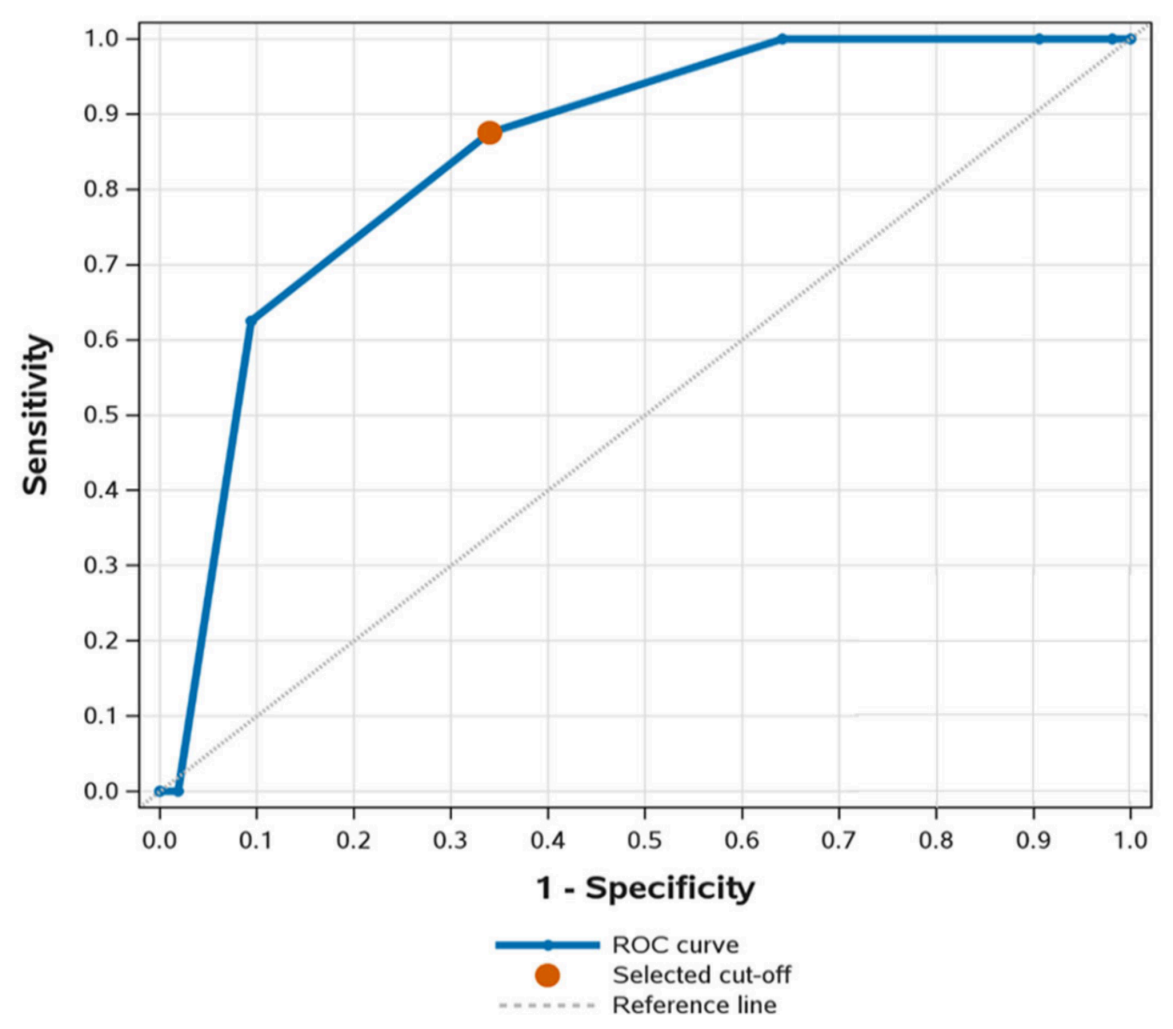
Prediction of pneumonitis using a threshold maximum score/score at pneumonitis of four points, illustrated by receiver operating characteristic (ROC) curve. AUC was 0.85 (95% confidence interval: 0.73–0.97).

The performance of the scoring system varied by threshold. The highest Youden index (0.535) was observed at a threshold score of four points. At this threshold, sensitivity was 87.5%, specificity was 66.0%, the positive predictive value was 28.0%, and the negative predictive value was 97.2% (Table 4 and Figure 3). Seven of the eight cases of pneumonitis were detected. A threshold score of five points yielded a similar Youden index (0.531); sensitivity, specificity, positive predictive value, and negative predictive value were 62.5%, 90.6%, 50.0%, and 94.1%, respectively (Table 4). Thus, compared with the threshold of four points, the threshold score of five points reduced sensitivity but substantially increased specificity. Thus, four points was more consistent with screening and early warning, whereas five points may be more suitable when greater specificity is desired.

**Figure 4:**
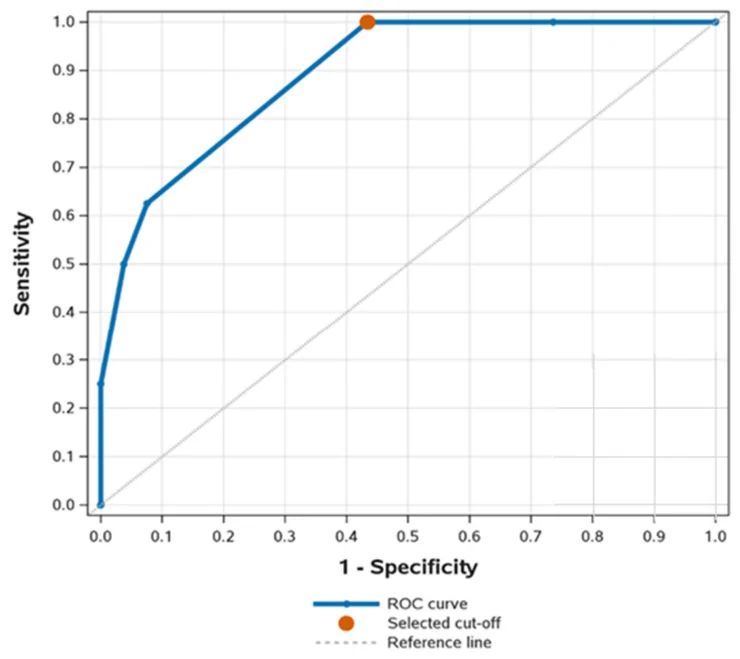
Prediction of pneumonitis using a threshold of two points for increase from baseline, illustrated by the receiver operating characteristic (ROC) curve. AUC was 0.89 (95% confidence interval: 0.80–0.99).

In addition to the primary maximum score or at the time of pneumonitis score, the difference between baseline and maximum scores (max_diff) was investigated as a supportive exploratory endpoint. Max_diff showed numerically stronger discrimination than the maximum score: with an AUC of 0.89 (95% confidence interval of 0.80 to 0.99). This AUC also exceeded the prespecified benchmark of 0.70 (*p* < 0.001). The optimal threshold identified by the Youden criterion was an increase of at least two points (Youden index = 0.566), with sensitivity of 100% and specificity of 56.6% (Table 5 and Figure 4). A more stringent increase of at least three points had higher specificity (92.5%), lower sensitivity (62.5%), and a similar Youden index of 0.550. However, the paired DeLong comparison of the primary score and max_diff did not show statistically superior discrimination for max_diff (*p* = 0.265). Therefore, max_diff should be interpreted as exploratory rather than as a replacement for the primary score. Sensitivities, specificities, and Youden indices, as well as the positive predictive and negative predictive values, for all threshold scores are summarized in Table 5.

Moreover, a total of sixteen patient-, cancer-, and treatment-related characteristics were evaluated for potential associations with the occurrence of radiation pneumonitis. The corresponding findings are shown in Table 6. No statistically significant characteristic was observed. Because only eight pneumonitis events occurred, these exploratory analyses had limited power and should not be interpreted as evidence that the investigated characteristics are of no relevance.

Patient satisfaction with the scoring system was an important second objective of the POLCAR trial. Questionnaires were available from 56 patients (91.8%). The other five patients did not complete the questionnaire without any reason given. To be considered satisfied, at least 16 of the maximum possible 28 points were required, representing a mean score of 4.0 or more points. Three of the 56 patients (5.4%) were not satisfied with the scoring system according to this definition. The corresponding values were 10, 11, and 15 points. In 20 patients (35.7%), satisfaction was maximal (total score of 28 points, average score of 7.0 points). Moreover, 17 patients (30.4%) had total scores of 24 to 27 points (average scores of 6.00 to 6.75 points), nine patients (16.1%) had total scores of 20 to 23 points (average scores of 5.00 to 5.75 points), and seven patients (12.5%) had total scores of 16 to 19 points (average scores of 4.00 to 4.75 points). In addition, mean scores and standard deviations of the four items included in the questionnaire were calculated and are presented in Table 7. All mean scores were well above 4.0 points.

## 4. Discussion

Thoracic irradiation in lung cancer patients can lead to radiation pneumonitis, which may turn out to be a serious complication. Severe pneumonitis was reported to be associated with decreased survival at three years [13]. In most cases, radiation pneumonitis is successfully treated with corticosteroids, particularly when it is detected early. A special circumstance that needs to be considered in this context is the fact that the vast majority of pneumonitis cases occur not during the course of radiotherapy but only up to 5 months afterwards, when the patients are not under close monitoring [12]. Since physicians and other medical staff members who are not experienced with irradiated lung cancer patients may not associate the clinical symptoms to the previous radiotherapy, radiation pneumonitis is likely to be missed and mistaken for pneumonia or bronchitis. As a consequence, the patient receives antibiotics instead of corticosteroids. Pneumonitis may become more severe resulting in a worse prognosis. Thus, it is very important to diagnose radiation pneumonitis as soon as possible. The achievement of this goal may be facilitated with the availability of a simple scoring system that can be used by the patients themselves. In the future, the results of the scoring system will ideally be included in an app that tells the patient to contact a pulmonologist or radiation oncologist when indicated.

Such a scoring system was previously evaluated in a prospective trial [6]. According to presence and intensity of symptoms, scores of zero to nine points were assigned. Higher scores represented a greater symptom burden. The main goal of that trial was the identification of the most appropriate threshold score to discriminate between presence of radiation pneumonitis and no pneumonitis. A threshold of five points provided the highest accuracy (discrimination ability). Moreover, radiation pneumonitis was associated with an increase in the score by at least three points from baseline prior to the start of irradiation [6]. However, several studies found that elderly patients have a significantly higher risk of radiation pneumonitis [25,26,27,28,29]. Therefore, it was unclear whether the threshold scores of the previously evaluated scoring system were valid also for this age group.

These considerations led to the current POLCAR trial, which was performed in a cohort of patients aged 65 years or older. According to its results, a threshold of four points appeared most appropriate for screening and early detection of radiation pneumonitis. This differs from the five points identified as optimal in the previous trial without age restriction [6]. With a threshold of four points, seven of the eight pneumonitis cases were identified, and the negative predictive value was high. However, the moderate specificity and low positive predictive value indicate that a score of at least four points should trigger further clinical assessment rather than be used as a standalone diagnostics criterion. A threshold of five points might represent a clinically useful alternative when specificity is prioritized. This threshold may be preferable when the purpose is to reduce false-positive alerts or to support diagnostic confirmation rather than early detection. External validation is required before the four-point threshold can be considered definitive for older patients.

In addition, the role of the difference between maximum score and baseline score was investigated. For this approach, the most appropriate threshold identified by the Youden criterion was an increase of at least two points. When applying this threshold, all pneumonitis cases were detected (sensitivity of 100%). Thus, this approach represented an attractive early warning threshold while maintaining a specificity of 56.6%. The threshold of two points was different from the previous prospective trial, where an increase in three points was considered most appropriate [6]. A more stringent threshold of an increase from baseline by three points in the present POLCAR trial improved specificity to 92.5% but reduced sensitivity, making it more suitable if additional diagnostic investigations were triggered by a positive result. An increase from baseline showed a numerically stronger discrimination when compared to the absolute score (AUC 0.89 vs. 0.85). However, its practical value must be weighed against its additional complexity. Of note, exploratory comparison of the AUCs of the absolute score and the increase from baseline with a paired DeLong test for two correlated ROC curves did not show evidence of statistically superior discrimination (*p* = 0.26). The absolute score is considered preferable for routine implementation because it is simpler and does not require a baseline reference value. The increase form baseline may serve as a refined risk-stratification measure when reliable data regarding baseline symptoms are available, particularly in patients with pre-existing respiratory symptoms. External validation is required to determine whether the small gain in discrimination justifies the added complexity.

Moreover, a total of sixteen characteristics were evaluated for associations with radiation pneumonitis. However, a significant association trends were not observed for any of these. This finding should be interpreted cautiously because the number of pneumonitis events was small. The absence of statistically significant associations does not exclude clinically relevant effects. Moreover, this finding was inconsistent with the results of other studies. At least for some of the characteristics investigated in the POLCAR trial, a significant association with the occurrence of radiation pneumonitis or a corresponding trend was previously described. These characteristics included cardiovascular disease, chronic obstructive lung disease, history of autoimmune disease, reduced pulmonary function, and additional systemic treatment [12,25,26,27,28,29,30]. The inconsistency may be explained by the fact that the studies except our own previous retrospective study did not specifically focus on elderly patients. Moreover, in our retrospective study, no factor was significantly associated with radiation pneumonitis; autoimmune disease and cardiovascular disease showed only trends [27].

The low number of events represents a significant limitation of the POLCAR trial. The clinical trial protocol assumed a pneumonitis rate of 30% whereas only eight cases of pneumonitis were observed. This limited the precision of threshold-specific operating characteristics and reduced power for exploratory risk-factor analyses. In addition, since cut-offs were selected and evaluated in the same dataset, the proposed thresholds should be regarded as preliminary and require external validation. Another limitation is the fact that 31 of the 61 patients were previously included in the prospective PARALUC trial. However, the design and the eligibility criteria of both trials were almost identical, and patients of the previous PARALUC trial were treated at institutions that participated in the current POLCAR trial. Therefore, the risk of an associated selection was considered low and acceptable. The fact that the number of pack-years (smoking history) was not assessed represents another limitation of the current trial, particularly because smoking status was reported to be a risk factor for radiation pneumonitis in a practice guideline from the European Society for Radiotherapy and Oncology (ESTRO) [31]. Another limitation is the fact that biomarkers, such as EGFR and PD-L1, were not assessed in this study. Since all patients received conventionally fractionated radiotherapy, the results of the POLCAR trial cannot be transferred to other radiotherapy approaches such as stereotactic body radiation therapy (SBRT) or hyper-fractionated irradiation, which are used to treat elderly lung cancer patients [32,33,34,35,36,37,38,39]. Despite these limitations, the primary objective of this trial (AUC >0.7) was clearly met. However, validation of its results should be performed in an additional prospective trial.

## 5. Conclusions

The investigated scoring system showed a good discrimination and a low dissatisfaction rate of only 5.4%. A threshold of four points appeared suitable for screening and early detection of pneumonitis in elderly patients. Moreover, an increase from baseline by two points provided high accuracy (discrimination ability). Since baseline scores were not required for the absolute score, the use of this approach appeared preferable for routine implementation when compared to the difference between baseline and maximum scores. The fact that the appropriate threshold score of four points was different from the score previously identified in a trial without age restriction illustrated the need for a separate tool for elderly patients. This aspect was further supported by different optimal thresholds regarding the increase from baseline, namely two points in the current POLCAR trial versus three points in the previous trial performed in patients of any age. Despite the limitations of the POLCAR trial, its results should be considered an important step toward the development of an app that can be used by the patients themselves and, consequently, toward an earlier detection of radiation pneumonitis.

## Figures and Tables

**Table 1:** Symptoms and corresponding scores, ranging between zero and nine points [6,18,19].

Symptom	Severity (Rated by Patients)	Score (Points)
Cough	No	0
	Yes, a little/sometimes	1
	Yes, moderate/regularly	2
	Yes, severe/permanently	3
Shortness of breath	No	0
	Yes, during intense exertion (e.g., climbing stairs)	1
	Yes, during mild exertion (e.g., walking on flat ground)	2
	Yes, at rest	3
Temperature	No	0
	Yes, between 37.6 and 38.0 °C	1
	Yes, between 38.1 and 39.0 °C	2
	Yes, higher than 39.0 °C	3

**Table 2:** Distribution of patient-, cancer-, and treatment-related characteristics of the 61 patients included in the analyses with respect to primary and secondary objectives.

Characteristic	Number of Patients (%)
Gender	
Female	23 (37.7)
Male	38 (62.3)
Age	
≤73 years	35 (57.4)
>73 years	26 (42.6)
ECOG performance score	
0–1	51 (83.6)
2–3	10 (16.4)
Body-mass index	
<30 kg/m^2^	53 (86.9)
≥30 kg/m^2^	8 (13.1)
Primary tumor stage	
T 1–2	16 (26.2)
T 3–4	43 (70.5)
Not specified	2 (3.3)
Lymph node status	
N 0–1	18 (29.5)
N 2–3	43 (70.5)
Metastatic disease	
M 0	49 (80.3)
M 1	12 (19.7)
AJCC stage	
I–II	6 (9.8)
III–IV	55 (90.2)
Histology	
Non-small-cell lung cancer	50 (82.0)
Small-cell lung cancer	11 (18.0)
Upfront resection	
No	55 (90.2)
Yes	6 (9.8)
Systemic treatment prior to and/or during the radiotherapy course	
No	5 (8.2)
Yes	56 (91.8)
Consolidation systemic treatment	
No	31 (50.8)
Yes	30 (49.2)
Total radiation dose	
≤60 Gy	30 (49.2)
>60 Gy	31 (50.8)
FEV1 prior to radiotherapy	
<1.6 L	29 (47.5)
≥1.6 L	29 (47.5)
Not available	3 (4.9)
Significant cardiovascular disease	
No	34 (55.7)
Yes	27 (44.3)
Chronic obstructive lung disease	
No	43 (70.5)
Yes	18 (29.5)
History of autoimmune disease	
No	56 (91.8)
Yes	5 (8.2)

AJCC: American Joint Committee on Cancer; ECOG: Eastern Cooperative Oncology Group.

**Table 3:** Baseline scores, maximum scores, and differences between both scores in the entire cohort, in patients developing pneumonitis, and in patients without pneumonitis. The *p*-values were obtained from two-sided Wilcoxon rank-sum (Mann–Whitney) tests with tie correction.

Variable	Entire Cohort Median (Range) Mean (SD)	No Pneumonitis Median (Range) Mean (SD)	Pneumonitis Median (Range) Mean (SD)	*p*-Value
Baseline score	2 (0–4) 1.62 (1.00)	2 (0–4) 1.70 (1.01)	1 (0–2) 1.13 (0.83)	0.14
Maximum score	3 (0–6) 3.18 (1.27)	3 (0–6) 2.98 (1.22)	5 (3–5) 4.50 (0.76)	**0.001**
Difference between baseline and maximum scores	2 (0–5) 1.56 (1.27)	1 (0–4) 1.28 (1.03)	3.5 (2–5) 3.38 (1.30)	**<0.001**

SD: standard deviation; bold *p*-values indicate statistical significance.

**Table 4:** Estimation of sensitivities, specificities, Youden indices, positive predictive values, and negative predictive values stratified by specific cut-offs for the maximum score.

Threshold for the Maximum Score	Sensitivity	Specificity	Youden Index	Positive Predictive Value	Negative Predictive Value
0 points	1.000	0.000	0.000	0.131	–
1 point	1.000	0.019	0.019	0.133	1.000
2 points	1.000	0.094	0.094	0.143	1.000
3 points	1.000	0.358	0.358	0.190	1.000
4 points	0.875	0.660	0.535	0.280	0.972
5 points	0.625	0.908	0.531	0.500	0.941
6 points	0.000	0.981	−0.019	0.000	0.867

**Table 5:** Estimation of sensitivities, specificities, Youden indices, and positive and negative predictive values, stratified by cut-offs for the difference between baseline and maximum score.

Threshold for the Maximum Score	Sensitivity	Specificity	Youden Index	Positive Predictive Value	Negative Predictive Value
0 points	1.000	0.000	0.000	0.131	–
1 point	1.000	0.264	0.264	0.170	1.000
2 points	1.000	0.566	0.566	0.258	1.000
3 points	0.625	0.925	0.550	0.556	0.942
4 points	0.500	0.962	0.462	0.667	0.927
5 points	0.250	1.000	0.250	1.000	0.898

**Table 6:** Numbers and rates of pneumonitis with regard to patient-, cancer-, and treatment-related characteristics of the 61 patients. *p*-values were obtained with Fisher’s exact test.

Characteristic	Pneumonitis, *n* (%)	*p*-Value
Gender		1.00
Female	3 (13.0)	
Male	5 (13.2)	
Age		1.00
≤73 years	5 (14.3)	
>73 years	3 (11.5)	
ECOG performance score		0.12
0–1	5 (9.8)	
2–3	3 (30.0)	
Body-mass index		1.00
<30 kg/m^2^	7 (13.2)	
≥30 kg/m^2^	1 (12.5)	
Primary tumor stage		0.67
T 1–2	3 (18.8)	
T 3–4	5 (11.6)	
Lymph node status		1.00
N 0–1	2 (11.1)	
N 2–3	6 (14.0)	
Metastatic disease		1.00
M 0	7 (14.3)	
M 1	1 (8.3)	
AJCC stage		1.00
I–II	1 (16.7)	
III–IV	7 (12.7)	
Histology		1.00
Non-small-cell lung cancer	7 (14.0)	
Small-cell lung cancer	1 (9.1)	
Upfront resection		1.00
No	8 (14.5)	
Yes	0 (0.0)	
Systemic treatment prior to and/or during the radiotherapy course		1.00
No	0 (0.0)	
Yes	8 (14.3)	
Consolidation systemic treatment		0.47
No	3 (9.7)	
Yes	5 (16.7)	
Total radiation dose		1.00
≤60 Gy	4 (13.3)	
>60 Gy	4 (12.9)	
FEV1 prior to radiotherapy		0.25
<1.6 L	2 (6.9)	
≥1.6 L	6 (20.7)	
Significant cardiovascular disease		1.00
No	4 (11.8)	
Yes	4 (14.8)	
Chronic obstructive lung disease		0.42
No	7 (16.3)	
Yes	1 (5.6)	
History of autoimmune disease		0.12
No	6 (10.7)	
Yes	2 (40.0)	

AJCC: American Joint Committee on Cancer; ECOG: Eastern Cooperative Oncology Group.

**Table 7:** Patient satisfaction with the scoring system.

Scoring System Rated	Mean Score	Standard Deviation
Comprehensible	6.32	1.28
Safe (feeling of safety)	5.77	1.52
Easy to use	6.36	1.26
Helpful	5.57	1.62

## Data Availability

Further information is available at clinicaltrials.gov (identifier: NCT06480734, posted on 28 June 2024).

## Abbreviations

The following abbreviations are used in this manuscript:

AJCC	American Joint Committee on Cancer
AUC	Area Under the Curve
BMI	Body-Mass Index
ECOG	Eastern Cooperative Oncology Group
EGFR	Epidermal Growth Factor Receptor
ESTRO	European Society for Radiotherapy and Oncology
FEV1	Forced Expiratory Volume in 1 s
HeAT	Health Advancing Technology for Elderly
NSCLC	Non-Small-Cell Lung Cancer
PD-L1	Programmed Death-Ligand 1
ROC	Receiver Operating Characteristic
SBRT	Stereotactic Body Radiation Therapy
SCLC	Small-Cell Lung Cancer
